# The Bacterial Sequential Markov Coalescent

**DOI:** 10.1534/genetics.116.198796

**Published:** 2017-03-02

**Authors:** Nicola De Maio, Daniel J. Wilson

**Affiliations:** * Institute for Emerging Infections, Oxford Martin School, University of Oxford, Oxford, OX1 3PA, United Kingdom; † Nuffield Department of Medicine, University of Oxford, Oxford, OX1 3PA, United Kingdom; ‡ Wellcome Trust Centre for Human Genetics, University of Oxford, Oxford, OX1 3PA, United Kingdom

**Keywords:** bacterial evolution, recombination, coalescent, simulations, ABC

## Abstract

Bacteria can exchange and acquire new genetic material from other organisms directly and via the environment. This process, known as bacterial recombination, has a strong impact on the evolution of bacteria, for example, leading to the spread of antibiotic resistance across clades and species, and to the avoidance of clonal interference. Recombination hinders phylogenetic and transmission inference because it creates patterns of substitutions (homoplasies) inconsistent with the hypothesis of a single evolutionary tree. Bacterial recombination is typically modeled as statistically akin to gene conversion in eukaryotes, *i.e.*, using the coalescent with gene conversion (CGC). However, this model can be very computationally demanding as it needs to account for the correlations of evolutionary histories of even distant loci. So, with the increasing popularity of whole genome sequencing, the need has emerged for a faster approach to model and simulate bacterial genome evolution. We present a new model that approximates the coalescent with gene conversion: the bacterial sequential Markov coalescent (BSMC). Our approach is based on a similar idea to the sequential Markov coalescent (SMC)—an approximation of the coalescent with crossover recombination. However, bacterial recombination poses hurdles to a sequential Markov approximation, as it leads to strong correlations and linkage disequilibrium across very distant sites in the genome. Our BSMC overcomes these difficulties, and shows a considerable reduction in computational demand compared to the exact CGC, and very similar patterns in simulated data. We implemented our BSMC model within new simulation software FastSimBac. In addition to the decreased computational demand compared to previous bacterial genome evolution simulators, FastSimBac provides more general options for evolutionary scenarios, allowing population structure with migration, speciation, population size changes, and recombination hotspots. FastSimBac is available from https://bitbucket.org/nicofmay/fastsimbac, and is distributed as open source under the terms of the GNU General Public License. Lastly, we use the BSMC within an Approximate Bayesian Computation (ABC) inference scheme, and suggest that parameters simulated under the exact CGC can correctly be recovered, further showcasing the accuracy of the BSMC. With this ABC we infer recombination rate, mutation rate, and recombination tract length of *Bacillus cereus* from a whole genome alignment.

BACTERIAL whole-genome sequencing has rapidly replaced multilocus sequence typing for population analyses of bacterial pathogens thanks to its fast and cost-effective provision of higher resolution genetic information (Didelot *et al.* 2012; Wilson 2012). Methods using genomic data to infer epidemiological, phylogeographic, phylodynamic, and evolutionary patterns are often hampered by recombination (*e.g.*, Schierup and Hein 2000; Posada and Crandall 2002), and the bacterial setting is no exception (Hedge and Wilson 2014). Recombination causes different sites in the genome to have different inheritance histories. For these reasons, in recent years many methods have been proposed to measure, identify, and account for bacterial recombination (*e.g.*, Didelot and Falush 2007; Marttinen *et al.* 2008; Tang *et al.* 2009; Didelot *et al.* 2010; Marttinen *et al.* 2012; Croucher *et al.* 2014; Didelot and Wilson 2015). Among these, simulators of bacterial evolution (*e.g.*, Didelot *et al.* 2009b; Mostowy *et al.* 2014; Brown *et al.* 2015) have been used for parameter inference and hypothesis testing (Fearnhead *et al.* 2005; Fraser *et al.* 2005; Wilson *et al.* 2009; Ansari and Didelot 2014), and for benchmarking (*e.g.*, Falush *et al.* 2006; Didelot and Falush 2007; Turner *et al.* 2007; Buckee *et al.* 2008; Marttinen *et al.* 2012; Hedge and Wilson 2014).

Simulating bacterial evolution poses specific difficulties as the process of bacterial recombination is very different to that of other organisms. Eukaryotic recombination is predominantly modeled as a cross-over process, with recombination events breaking a chromosome into two parts with different ancestries (Figure 1). While it is possible to simulate eukaryotic evolution with recombination forward in time (Peng and Kimmel 2005; Carvajal-Rodríguez 2008; Hernandez 2008; Arenas 2013), coalescent-based (Kingman 1982) backward in time models (Hudson 1983; Griffiths and Marjoram 1997; Wiuf and Hein 1999) are usually more computationally efficient (*e.g.*, Hudson 2002; Arenas and Posada 2007, 2010; Ewing and Hermisson 2010; Excoffier and Foll 2011). Yet, the coalescent with recombination itself may not be sufficiently fast when large genomic segments are considered (McVean and Cardin 2005). One of the reasons is that the structure describing the evolutionary history of all positions (the ancestral recombination graph, ARG) grows subexponentially with genome size and recombination rate (Wiuf and Hein 1999). For this reason, a faster approximation to the coalescent with recombination, the sequential Markov coalescent [SMC, see McVean and Cardin (2005), Marjoram and Wall (2006)] was proposed. Similar to the exact sequential form of the coalescent with recombination (Wiuf and Hein 1999), the SMC starts by considering one evolutionary tree at the left (*i.e.*, $5^{\prime }$) end of the sequence, and generates new trees affected by recombination as it moves toward the right ($3^{\prime }$) end. However, the SMC does not generate an ARG, but rather a sequence of local trees. The SMC makes the simplifying assumption that, if the local tree for the considered position is known, then all local trees to its left can be ignored when considering trees to its right. In fact, crossover recombination makes evolutionary histories less correlated as physical distance increases. This model has been extended to incorporate complex population history (Chen *et al.* 2009), and to have improved accuracy (Wang *et al.* 2014) and computational efficiency (Staab *et al.* 2015).

**Figure 1:**
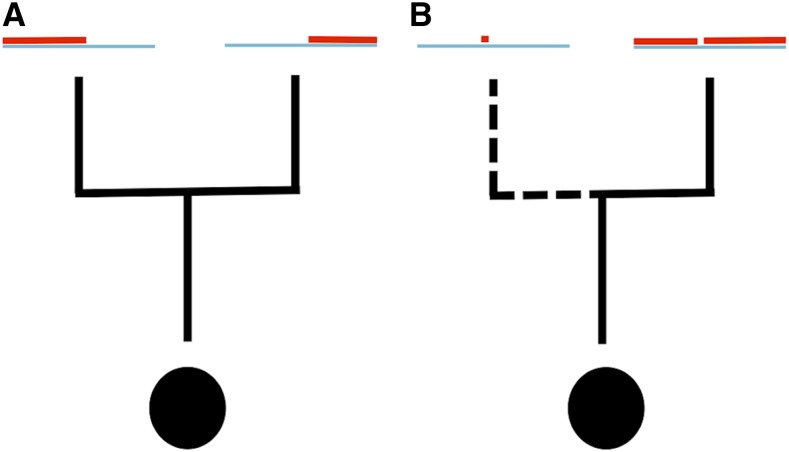
Graphical representation of eukaryotic and bacterial recombination models. Black circles represent sampled sequences, black lines are ancestral lineages (dashed if they represent bacterial recombination lineages). Blue segments represent the genome sequence, and red segments represent the portion of the genome that is ancestral to the particular lineage. (A) Crossover event: the entire genome to the left of the crossover site is inherited from one parent; the entire genome to the right is inherited from the other parent. (B) Gene conversion, or bacterial recombination: most of the genome is inherited from a single parent lineage, except a short segment.

Bacterial recombination is different from eukaryotic recombination (Smith *et al.* 1993, 2000), and is generally modeled like gene conversion: a bacterial recombination event imports only a small fragment of DNA from a donor genome, while most of the genome is inherited clonally (Figure 1). This results in sites very distantly located in the genome remaining very tightly linked genetically. In fact, a single genealogy, known as the clonal frame (Milkman and Bridges 1990), represents the evolutionary history of all nonrecombining sites, no matter how physically far they are from each other. So, methods for eukaryotic recombination cannot be applied to bacteria at genomic scales. While bacterial evolution can be simulated forward in time, backward in time coalescent methods are usually more efficient, and are generally based on the coalescent with gene conversion [CGC, see Wiuf and Hein (2000), and Figure 2A]. Recently, efficient methods implementing the CGC have been developed for simulating bacterial evolution (Didelot *et al.* 2009b; Brown *et al.* 2015). However, these approaches struggle to simulate whole genomes at high recombination rates (*e.g.*, requiring up to hours for one bacterial genome alignment with ρ>0.01, see Brown *et al.* 2015, and *Results*). Like the coalescent with crossover recombination, the CGC also generates large ARGs, and this contributes to the computational demand.

**Figure 2:**
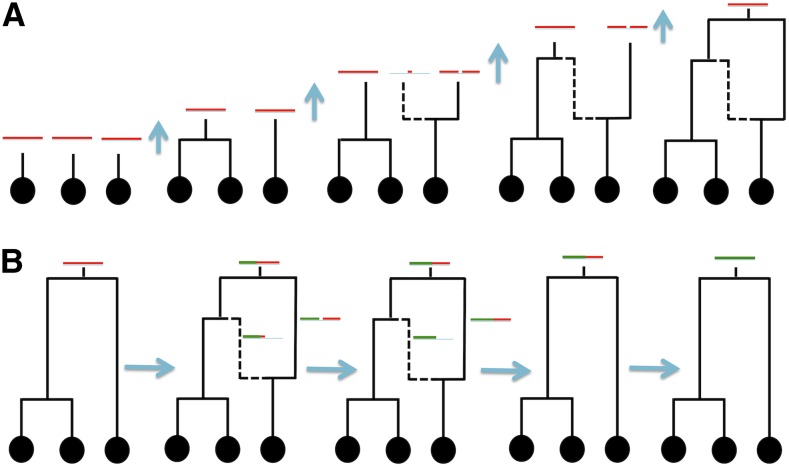
Graphical representation of the bacterial coalescent (CGC) and BSMC models. Black circles represent sampled genomes, black lines are ancestral lineages (continuous if they belong to the clonal frame, dashed otherwise). Red segments represent, for each extant lineage, the portion of the genome that is ancestral to any sampled descendent of that lineage. Time is considered backward from bottom to top, and mergers of lineages represent coalescent events. (A) Example of simulation under the CGC; recombination and coalescent events are simulated backward in time starting with one lineage per sample at the present. (B) Example of BSMC simulation: first a clonal frame is simulated; then the process moves left to right across the genome (which for simplicity is linear), and left portions of the genome are gradually forgotten (represented in green). The BSMC stops at each recombination start and end position; recombination events are forgotten at their end, but the clonal frame is never forgotten.

Here, we present a new approximation to the CGC (Figure 2B), inspired by the SMC, to efficiently and accurately model bacterial recombination. We model the clonal frame, and simulate the coalescent and recombination processes from one end of the genome to the other, conditioning on the clonal frame throughout. However, we ignore recombination events that occurred at distant, previously considered, positions. This approach differs from other approximations to the CGC (*e.g.*, Didelot *et al.* 2010; Ansari and Didelot 2014), as we can simulate entire genomes while allowing recombining lineages with overlapping ancestral material to coalesce with one another, and allowing recombination events to split the ancestral material of recombinant lineages. In fact, frequent recombination events can break down ancestral material intervals further and further, reducing them far below the expected length of an individual recombination interval. Ignoring these complexities leads to biases when considering elevated recombination rates (Didelot *et al.* 2010), and, by accounting for them, we aim to produce a model more faithful to the CGC. We call this model the bacterial sequential Markov coalescent (BSMC), which we implement within new simulation software called FastSimBac. FastSimBac is faster than previous methods (between one to two orders of magnitude for typical genome sizes and recombination rates). Also, by building on top of popular simulators ms (Hudson 2002) and MaCS (Chen *et al.* 2009), our software can simulate general evolutionary scenarios, allowing migration, speciation, demographic changes, recombination hotspots, and between-species recombination. We show that the BSMC can accurately approximate the exact CGC, and can be used to infer recombination parameters using Approximate Bayesian Computation (ABC). We demonstrate its utility by inferring *Bacillus cereus* recombination and mutation parameters from a whole genome alignment.

## Materials and Methods

### BSMC algorithm

We assume that a given set of parameters is specified *a priori*: *λ* is the mean length of a recombining segment, *G* is the total genome length, and *ρ* is the recombination rate. *λ* and *G* are measured in base pairs, while $\rho =2N_{\text{e}}r$ is the per-individual, per-generation, and per-base pair gene conversion initiation rate *r* scaled by twice the effective population size $N_{\text{e}}.$ Our BSMC algorithm crosses the genome from left to right, and discards most previous local trees, but always keeps track of, and conditions on, the clonal frame. The current local ARG $A\left(x_{\mathrm{cur}}\right)$ keeps track of all, and only the lineages with nonempty ancestral material to the right of $x_{\mathrm{cur}}.$ All lineages in $A\left(x_{\mathrm{cur}}\right)$ are possible targets of new recombination events and coalescent events. Recombination events and coalescent events are not allowed on forgotten lineages [not in $A\left(x_{\mathrm{cur}}\right)$]. To determine which lineage is in $A\left(x_{\mathrm{cur}}\right)$ and which is not, we record and update for each lineage *l* its ancestral material to the right of $x_{\mathrm{cur}}:a_{l}\left(x_{\mathrm{cur}}\right).$ One aim of the algorithm is to generate the sequence of local trees along the genome. For a given position $x_{\mathrm{cur}},$ the local (or marginal) tree $T\left(x_{\mathrm{cur}}\right)$ is the genealogy describing the inheritance history of site $x_{\mathrm{cur}}.$ $T\left(x_{\mathrm{cur}}\right)$ can be obtained from $A\left(x_{\mathrm{cur}}\right)$ by removing all branches that are not ancestral at $x_{\mathrm{cur}}.$ A graphical example of the algorithm is given in Supplemental Material, Figure S1 in File S1. More specifically, the BSMC algorithm proceeds as follows:

1. *Initialization*:*$x_{\mathrm{cur}}=0$* (current position, maximum is 1), and $T_{\mathrm{cf}}$ (the clonal frame) is simulated under the coalescent without recombination. The initial local ARG $A\left(x_{\mathrm{cur}}\right),$ and local tree $T\left(x_{\mathrm{cur}}\right),$ are set to $T\left(0\right)=A\left(0\right)=T_{\mathrm{cf}}.$ The ancestral material of every lineage *l* in A(0) is set to $a_{l}\left(0\right)=\left[x_{\mathrm{cur}},1\right]=\left[0,1\right],$ the whole genome. The list of recombination end points *E* (the right ends of recombination segments) is initialized as empty: E=().
2. *Position of new event*: The distance until the next potential recombination initiation (that occurs at position $x_{\mathrm{new}}$) is drawn according to an exponential distribution $\left(x_{\mathrm{new}}-x_{\mathrm{cur}}\right)∼\mathrm{Exp}\left[\left(\rho G/2\right)\bar{A}\left(x_{\mathrm{cur}}\right)\right],$ where $\bar{A}\left(x_{\mathrm{cur}}\right)$ is the sum of all branch lengths in $A\left(x_{\mathrm{cur}}\right),$ expressed in units of $2N_{\text{e}}$ generations. If $x_{\mathrm{new}}>E_{0},$ where $E_{0}$ is the first (and smallest) element of the list *E* of recombination end points (if *E* is empty then $E_{0}=\infty$), then the recombination initiation at $x_{\mathrm{new}}$ is cancelled, and the next considered position is set to $x_{\mathrm{new}}=E_{0};$ $E_{0}$ is then removed from *E*, and the next event becomes a recombination termination, so go to step 4. Otherwise, if $x_{\mathrm{new}}\geq 1$ terminate the algorithm, and if $x_{\mathrm{new}}<1$ the next event is a new recombination initiation at $x_{\mathrm{new}},$ so go to step 3.
3. *New recombination event*: sample a lineage *l* randomly from $A\left(x_{\mathrm{cur}}\right)$ proportionally to branch length. Then, sample a time *t* uniformly along the time spanned by *l*. The new recombination occurs at time *t* on branch *l*, and a new lineage $l^{\prime }$ is created, with its most recent end joining *l* at time *t*. A new coalescent time and coalescing lineage is sampled for $l^{\prime }$ conditional on $A\left(x_{\mathrm{cur}}\right)$ [under the algorithm of Wiuf and Hein (1999)]. The right end of the recombining interval $x_{\mathrm{end}}$ is sampled from the distribution $\left(x_{\mathrm{end}}-x_{\mathrm{new}}\right)∼\mathrm{Geom}\left(\lambda \right)/G,$ where Geom(λ) is the geometric distribution with mean *λ*. If $x_{\mathrm{end}}<1,$ it is added to *E* while keeping *E* sorted in increasing order. The new local ARG is defined as $A\left(x_{\mathrm{new}}\right)=A\left(x_{\mathrm{cur}}\right)\cup l^{\prime },$ and ancestral material of all lineages in $A\left(x_{\mathrm{new}}\right)$ is updated (ancestral material to the left of $x_{\mathrm{new}}$ is deleted). Any lineage with no ancestral material to the right of $x_{\mathrm{new}}$ is removed from $A\left(x_{\mathrm{new}}\right).$ The new local tree $T\left(x_{\mathrm{new}}\right)$ is defined from $A\left(x_{\mathrm{new}}\right)$ and is printed to file. The current position is updated: $x_{\mathrm{cur}}=x_{\mathrm{new}}.$ Return to step 2.
4. *Terminate a recombination event*: the new local ARG is initialized as $A\left(x_{\mathrm{new}}\right)=A\left(x_{\mathrm{cur}}\right).$ The ancestral material of all lineages in $A\left(x_{\mathrm{new}}\right)$ is updated (ancestral material to the left of $x_{\mathrm{new}}$ is deleted). Any lineage with no ancestral material to the right side of $x_{\mathrm{new}}$ is removed from $A\left(x_{\mathrm{new}}\right).$ The new local tree $T\left(x_{\mathrm{new}}\right)$ is defined from $A\left(x_{\mathrm{new}}\right)$ and is printed to file. The current position is updated: $x_{\mathrm{cur}}=x_{\mathrm{new}}.$ Return to step 2.

A large part of the complexity of the algorithm is attributable to the process of updating the ancestral material of lineages after a new recombination event is added to the local ARG. This step is described more in detail in File S1. Our algorithm and model differ from the approximation of the CGC used by Didelot *et al.* (2010) and Ansari and Didelot (2014); in fact, we allow recombinant lineages to be affected by further recombinations, and to coalesce with each other if their ancestral materials overlap. To increase realism, we use the first positions simulated by the algorithm (generally 10λ bases) as burn-in, that is, they are simulated but not considered part of the genome. While we simulate a linear genome, bacterial genomes are typically circular, so we assume that an arbitrary start position has been chosen. The version of the algorithm above conveys the basics of the model of within-population recombination; in our simulation software FastSimBac we have included many additional event types described in File S1: mutation, migration, speciation, demographic change, recombination hotspots, and between-species recombination.

### Performance testing

We simulated bacterial genome evolution under the coalescent with gene conversion using SimBac (Brown *et al.* 2015). We always simulated 50 contemporaneous samples. We performed simulations under four different recombination intensities: $\rho =2N_{\text{e}}r=0.001,0.002,0.005,0.01,$ with *ρ* the population-scaled per-generation per-base pair recombination initiation rate. We used four genome sizes: *G* = 1, 2, 5, and 10 Mbp, and mean recombination tract length λ=500. These values encompass a range of biologically relevant scenarios for bacteria (Vos and Didelot 2009; Didelot and Maiden 2010). We simulated 10 replicates for each combination of parameters, and, for each replicate, the simulated collection of local trees, and the clonal frame, were stored. Sequence data were generated from local trees using SeqGen (Rambaut and Grassly 1997) under an HKY85 model (Hasegawa *et al.* 1985) with transition/transversion rate ratio κ=3. Some of the parameter combinations were too computationally demanding for SimBac: (ρ=0.005, *G* = 10 Mbp), (ρ=0.01, *G* = 5 Mbp), (ρ=0.01, *G* = 10 Mbp). Every time we could run SimBac, we used its clonal frame as a fixed input for our software FastSimBac. The clonal frame is a major source of variation in sequence patterns between simulations (Ansari and Didelot 2014), so fixing the clonal frame, we reduce the variance in the difference of summary statistics between the two methods. Both the BSMC and the CGC assume that the clonal frame is generated by a standard coalescent process, so fixing it does not introduce biases, and gives better resolution to spot differences between models. For any scenario in which we could not run SimBac, the clonal frame was generated within FastSimBac. We used local trees from FastSimBac to generate genome alignments with SeqGen as before.

### ABC inference

We performed ABC with the local-linear regression approach (Beaumont *et al.* 2002) as implemented in the R package abc (Csilléry *et al.* 2012). We implemented and tested the performance of an ABC scheme based on the BSMC, using FastSimBac simulations to infer parameters from datasets themselves simulated under the CGC with SimBac. We used a uniform prior distribution over [0,0.005] for the recombination rate *ρ*, and over [10,1000] for the mean length *λ* of recombining intervals. The same priors were used for both simulating datasets and performing inference. The aim of the ABC analyses was to infer *ρ* and *λ*. For simplicity, the clonal frame simulated in SimBac was assumed to be known, as was the mutation rate θ=0.005. The clonal frame is an important confounding factor in real data analysis, which can be hard to estimate correctly (Hedge and Wilson 2014). However, including clonal frame inference in our ABC would make it too computationally demanding; also, fixing the clonal frame in this context allows us to focus on differences between the BSMC and the CGC. We simulated 1 Mbp alignments with 20 samples. For each true data set from SimBac, we simulated 10,000 datasets under the BSMC in FastSimBac. Only 1% of the simulations in FastSimBac were retained for parameter inference [the 1% with summary statistics most closest to true data, see Beaumont *et al.* (2002)]. We used two summary statistics: G4 (the proportion of incompatible sites) between neighboring SNPs, and G4 between SNPs at least 20 kbp away. Specifically, for the first summary statistic we counted the number of SNPs inconsistent with the first SNP occurring to their right; for the second summary statistic, for each SNP we selected the first SNP to its right at least 20 kbp away. We chose these summary statistics because G4 [and linkage disequilibrium (LD)] at short distances (h≪λ) is informative of the recombination rate *ρ* (the expected number of recombination events initiating or terminating within a short interval *h* is approximately proportional to 2hρ). On the other hand, G4 at long distances (h≫λ) is informative of the product ρλ (the expected number of recombination events affecting any of two distant bases is approximately proportional to 2ρλ). The approximately linear relationship between G4 and number of recombinations might not hold for extreme values of the parameter space, in which case this simple two-summary statistics ABC could have problems inferring *ρ* and *λ* (see Figure 2 in File S1).

We also used the ABC-MCMC inference scheme (Marjoram *et al.* 2003) on a real *B. cereus* genome alignment (Didelot *et al.* 2010; Ansari and Didelot 2014). We used uniform prior distributions on [0.0,0.25] for *ρ*, on [1,10000] for *λ*, and on [0.01,0.2] for *θ* (the per-base pair per-individual, and per-generation mutation rate scaled by $2N_{\text{e}}$). *ρ*, *λ* and *θ* are also the three parameters that we inferred. We simulated whole genome alignments of 13 samples and 5,240,935 bp, as for the real dataset. For this analysis we used more summary statistics than in the ABC above (seven instead of two), so to allow estimation of the mutation rate *θ*, to address potential limitations of the two previously considered summary statistics (see Figure S2 in File S1), and to address the potential impact of biological complexities on individual summary statistics. The seven summary statistics used are: number of polymorphic sites (observed value 629,942); G4 for consecutive SNPs (observed value 0.167) and for SNPs at least 2 kbp away (observed value 0.297); mean LD (measured as $r^{2}=\left[{\left(p_{\text{AB}}-p_{\text{A}}p_{\text{B}}\right)}^{2}/p_{\text{A}}\left(1-p_{\text{A}}\right)p_{\text{B}}\left(1-p_{\text{B}}\right)\right]$ where $p_{\text{A}}$ is the frequency of allele A in the first SNP, $p_{\text{B}}$ the frequency of B in the second SNP, and $p_{\text{AB}}$ the frequency of the AB haplotype) for consecutive SNPs (observed value 0.396) and for SNPs at least 2 kbp away (observed value 0.274); and mean number of haplotypes (considering a certain number of SNPs at the time) for pairs of consecutive SNPs (observed value 3.003) and for groups of four SNPs made of two pairs of consecutive SNPs, the two pairs being at a distance of at least 2 kbp. The number of SNPs, G4 and $r^{2}$ were also used as summary statistics by Ansari and Didelot (2014). We can simulate entire genomes (instead of SNP pairs as Ansari and Didelot (2014)) and so include summary statistics for groups of >2 SNPs. Due to the considerable computational demand, we fixed the clonal frame to that estimated and used by Didelot *et al.* (2010) and Ansari and Didelot (2014). However, recombination can cause errors in the estimate of the clonal frame, in particular of branch lengths (Hedge and Wilson 2014). In fact, with increasing recombination, and, in the absence of population structure, all genetic distances between samples are expected to converge to a common value. The consequent branch length errors can potentially bias inference, so we attempt to correct branch length errors within our ABC approach (see File S1). Lastly, to improve the realism of our model, we account for invariable sites. In fact, ∼1 out of every 6 bp (after removing sites with limited coverage) in the alignment are polymorphic, and a large proportion of the genome is expected to be coding; so, in principle, one would expect many homoplasies to occur simply due to multiple substitutions at the same site, and not necessarily requiring recombination events. Using back of the envelop calculations (see File S1) we estimated that around half (48.44%) of the genome is invariant and that the transition-transversion ratio is 5.21. We used these estimates as fixed values within an HKY (Hasegawa *et al.* 1985) substitution model with invariant sites, instead of the basic JC model (Jukes and Cantor 1969) implemented in our basic inference and in Ansari and Didelot (2014). This model, together with the local trees simulated by FastSimBac, was used in SeqGen to simulate the alignment from which summary statistics were extracted at each step of the ABC-MCMC. Each run consisted of 10,000 ABC-MCMC steps (of which 1000 were used as burn-in), and required between 2 weeks to 1 month with one processor.

### Data availability

FastSimBac is distributed as open source under the terms of the GNU General Public License, is available from https://bitbucket.org/nicofmay/fastsimbac, and is distributed as open source under the terms of the GNU General Public License.

## Results and Discussion

### Computational efficiency of BSMC

**Figure 3:**
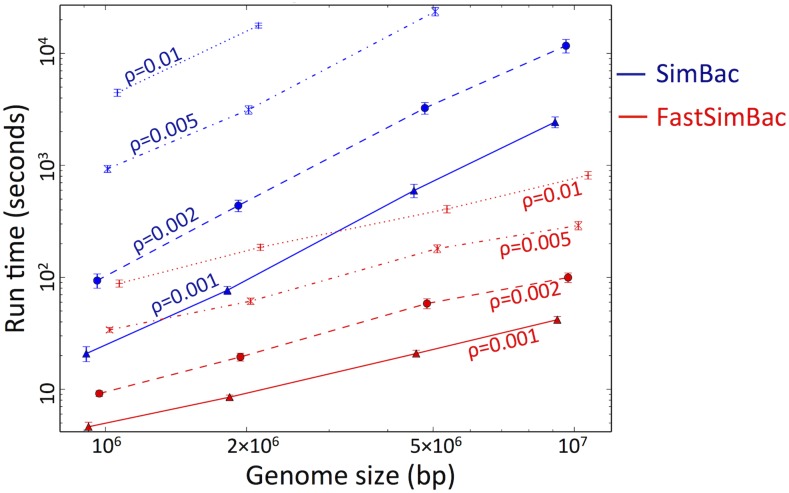
Comparison of computational demand between the bacterial sequential Markov coalescent (BSMC) and the coalescent with gene conversion (CGC). The BSMC implemented in FastSimBac is faster than the CGC implemented in SimBac. On the vertical axis is the time required to generate local trees per replicate (in seconds on a logarithmic scale). On the horizontal axis is the genome size (in base pair on a logarithmic scale). Red lines refer to FastSimBac, blue lines to SimBac. Each point is the mean over 10 replicates, and bars represent SEs of the mean. SimBac was not run for highest recombination rates and genome sizes due to time limitations.

FastSimBac substantially reduces computational demand for simulating bacterial genome evolution. Compared to SimBac (the most efficient software currently available), FastSimBac improves speed by ∼1 order of magnitude for low recombination rate (ρ=0.001) and genome size ($10^{6}$ bp), and up to two orders of magnitude for elevated recombination rate (ρ=0.01) and genome size ($10^{7}$ bp) (Figure 3). Further, FastSimBac allows simulation of scenarios with both high recombination rate and genome size, which are currently out of reach of other methods due to excessive requirements for time and RAM. The performance of FastSimBac relative to the CGC improves as we increase either genome size or recombination rate (Figure 3). The running time required appears linear with genome size for FastSimBac, while not for SimBac. Another benefit of FastSimBac is that, by avoiding the generation of a global ARG, it has small RAM usage, allowing to run multiple simulations in parallel. The computational demand of FastSimBac also appears approximately linear in the number of samples (Figure S3 in File S1).

### Accuracy of the BSMC

**Figure 4:**
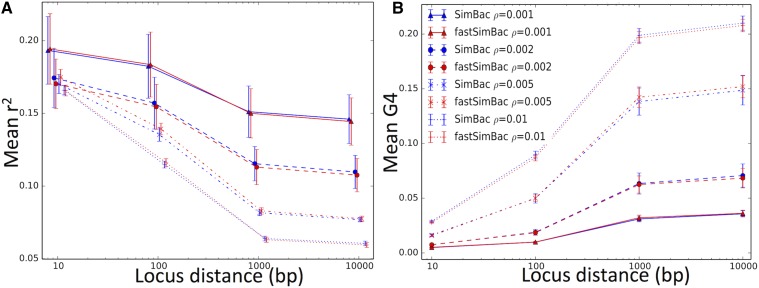
Comparison of LD and site incompatibility between the BSMC and the CGC. The BSMC generates patterns of LD (measured as $r^{2}$) and pairwise genetic incompatibility between sites (G4) very similar to the CGC. On the horizontal axis is the base pair distance between SNPs at which LD and G4 are measured. $r^{2}$ is calculated as $\left[{\left(p_{\text{AB}}-p_{\text{A}}p_{\text{B}}\right)}^{2}/p_{\text{A}}\left(1-p_{\text{A}}\right)p_{\text{B}}\left(1-p_{\text{B}}\right)\right],$ and G4 (the four-gamete test) is one if a SNP pair is incompatible and zero otherwise. For each distance *d*, and for any SNP *x*, LD and G4 are calculated between *x* and the first SNP at least *d* base pair to the right of *x*. Red lines refer to FastSimBac, blue lines to SimBac, and different point and line styles refer to different recombination rates (see legend). Genome length is 1 Mbp. Each point is the mean over 20 replicates, and bars are SEM. (A) Genome-wide mean LD. (B) Genome-wide mean G4.

Next, we compared the simulated patterns of genetic variation and local tree features between the exact CGC simulated under SimBac, and the BSMC simulated with FastSimBac. LD (measured as $r^{2}$), as expected, decreases considerably with increasing recombination rate (Figure 4), while the opposite holds for pairwise genetic incompatibility between sites (the four-gamete test, G4). There is substantial variation across different replicates in mean LD, probably because each replicate has a distinct clonal frame, and the clonal frame influences site patterns across the whole genome. LD and G4 at 1 kbp scales are already very close to those at longer distances, suggesting that a distance of 2λ is sufficient to reach nearly as much LD as any arbitrary distance. Most importantly, values simulated under the BSMC mimic closely those simulated under the CGC, suggesting that, even at high recombination rates and short distances, the BSMC is a very accurate approximation (Figure 4). Similar results are also observed at different genome sizes (Figure S4 in File S1).

**Figure 5:**
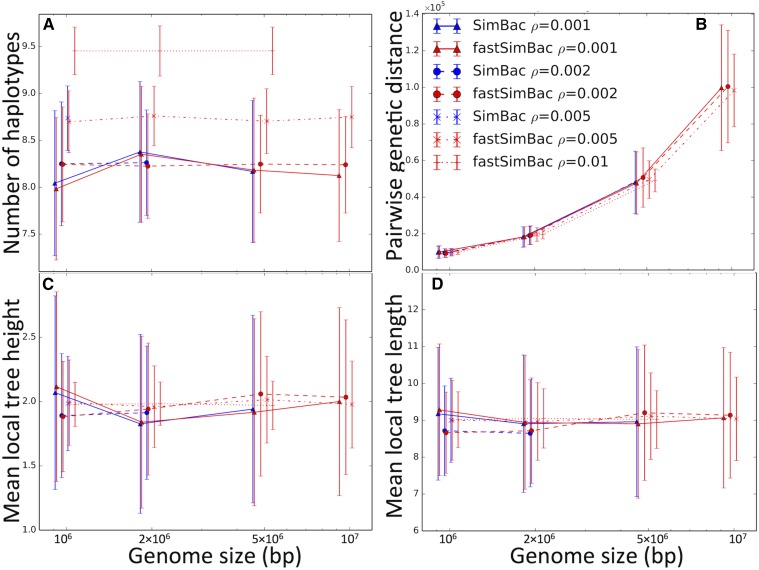
Comparison of simulated patterns between the BSMC and the CGC. Bacterial evolution simulated under the BSMC generates very similar patterns to the exact CGC. (A) Genome-wide mean number of simulated haplotypes over nonoverlapping sliding windows of 10 SNPs; (B) Mean pairwise genetic distance between samples; (C) Mean local tree height; (D) Mean local tree size (sum of all branch lengths). On the horizontal axis is genome size in base pair and on logarithmic scale. Red lines refer to FastSimBac, blue lines to SimBac, and different line and dot styles indicate different recombination rates (see legend). Each point is the mean over 50 replicates, and bars are SDs. SimBac and FastSimBac were not run for the highest recombination rates and genome sizes due to time and memory limitations.

As expected, the number of haplotypes in nonoverlapping windows of 10 SNPs increases with recombination rate (Figure 5A), and, again, the BSMC very closely mimics the CGC. The genomic variation in number of haplotypes (Figure S5A in File S1) is very slightly underestimated, probably because long-range correlations in local trees (after conditioning on the clonal frame) are ignored in the BSMC, while present in the CGC. The mean pairwise genetic distances between samples are unaffected by recombination and by the model used for simulations (Figure 5B), but recombination does affect their variance (Figure S5B in File S1) because it tends to break down the relatedness of samples. Again, both patterns in the CGC are very closely approximated by the BSMC. Mean local tree height (Figure 5C) and mean local tree size (total sum of the branch lengths, Figure 5D), are highly variable depending on the simulated clonal frame, and are not strongly affected by the simulation parameters, nor by our BSMC approximation.

### BSMC-based ABC inference

**Figure 6:**
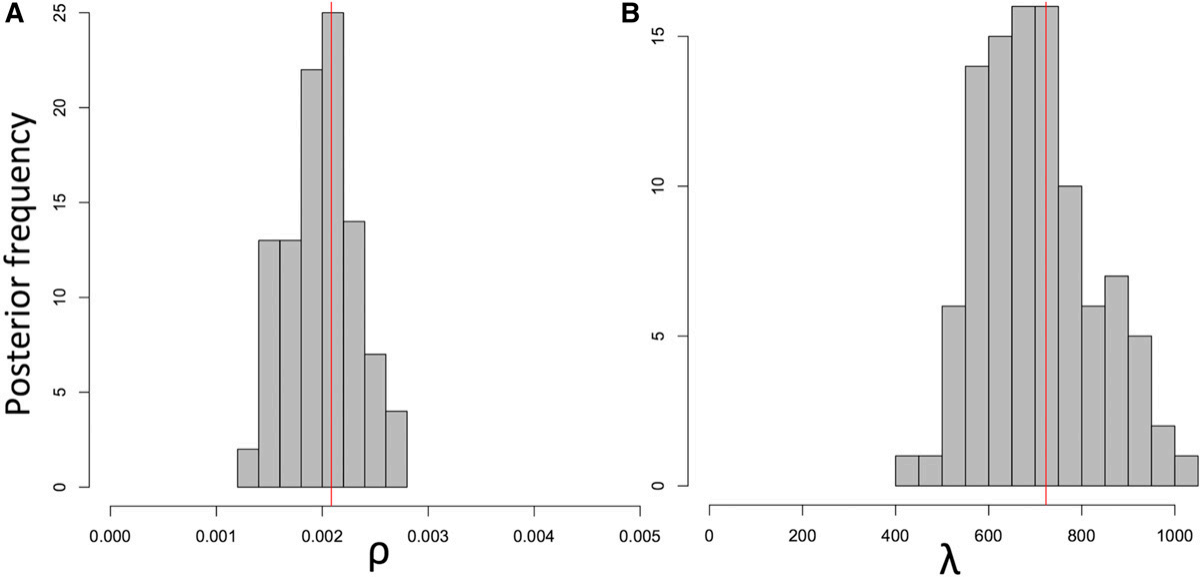
Accurate inference of recombination parameters with the BSMC-based ABC. Recombination parameters simulated under the exact CGC (red vertical lines) were reconstructed using simulations under the BSMC within an ABC inference scheme. Inference from another independent ABC run is shown in Figure S6 and File S1. (A) Posterior distribution of *ρ*. (B) Posterior distribution of *λ*.

We investigated the accuracy and applicability of the BSMC approximation by performing ABC inference. First, we reconstructed parameters simulated under the exact CGC. We use two summary statistics based on G4, the pairwise genetic incompatibility between sites (see *Materials and Methods*). Although we simulated datasets under the exact CGC, and performed ABC simulations under a different model (the BSMC), inference was accurate. The 95% posterior confidence intervals for the population-scaled recombination rate *ρ*, and the mean length of recombining intervals *λ* contain the simulated values in both our replicates (Figure 6 and Figure S6 in File S1). This suggests that the BSMC can be used for accurately inferring bacterial evolutionary parameters. However, the elevated computational demand of this ABC approach keeps us from performing a more thorough simulation study. Also, here we assume that the exact clonal frame is known, and focus on differences between the BSMC and the CGC; this is not usually true for real datasets, where clonal frame imprecision could likely lead to higher inference error.

As an additional example of the applicability of the BSMC and of FastSimBac, we used ABC-MCMC (Marjoram *et al.* 2003) to infer *ρ*, *λ*, and the scaled mutation rate *θ* for the *B. cereus* bacterial group. Bacteria of the *B. cereus* group mostly live in the soil, feeding on dead organic matter, but they can occasionally infect humans and cause a range of diseases, from food poisoning to deadly anthrax (Arnesen *et al.* 2008). Disagreement has been found between *B. cereus* species designation and MLST clade structure and population history, probably due to the contribution of plasmids and genetic recombination to the bacterial phenotype (Priest *et al.* 2004; Sorokin *et al.* 2006; Didelot *et al.* 2009a; Zwick *et al.* 2012). Furthermore, analyses of MLST data have shown discordant results regarding the prevalence of recombination relative to mutation in *B. cereus*. Estimates range from ρ/θ≈0.05 (Hanage *et al.* 2006), to ρ/θ≈0.2 (Didelot *et al.* 2009a), to ρ/θ≈0.3 (Didelot and Falush 2007), up to ρ/θ≈2 (Pérez-Losada *et al.* 2006), leading to a state of uncertainty regarding the contribution of recombination to *B. cereus* evolution. Improving our understanding of recombination in *B. cereus* would help us recognize the effect of homologous recombination on epidemiological inference and species delimitation (Didelot and Maiden 2010), and predict the acquisition and spread of infectivity and resistance factors (Perron *et al.* 2012). Genome-wide data from multiple strains provide a greater opportunity to study recombination. Here, we consider the genome alignment described in Didelot *et al.* (2010) and Ansari and Didelot (2014) comprising 13 genomes from the *B. cereus* group. Didelot *et al.* (2010) performed MCMC inference on this dataset using an approximate coalescent model with bacterial recombination (the ClonalOrigin model) that did not allow recombinant lineages to be affected by further recombination, nor recombinant lineages to coalesce with one another. They inferred a mean recombination tract length of λ=171 bp with interquartile range [168,175], and ρ/θ=0.21 with interquartile range [0.20,0.23]. Ansari and Didelot (2014) used a model similar to ClonalOrigin within an ABC-MCMC approach, and accounted for the propensity for lineages to recombine more with closely related lineages than with distantly related ones. They inferred ρ=0.077 with confidence interval $CI_{\rho }=\left[0.036,0.127\right],$ λ=152 bp with $CI_{\lambda }=\left[74,279\right],$ and θ=0.0528 with $CI_{\theta }=\left[0.0437,0.0640\right].$ The ClonalOrigin model employed by these methods approximates the coalescent with gene conversion, but less closely than the BSMC. In fact, the ClonalOrigin model leads to overestimation of *ρ* under recombination and mutation rates relevant to this scenario (Didelot *et al.* 2010). Our BSMC-based ABC-MCMC approach instead allows recombination events to split the ancestral material of recombinant lineages. Furthermore, in contrast to both these previous analyses, we account for differences in transition and transversion rates, for invariant sites, and for biases in branch length estimation (see *Materials and Methods* and File S1).

**Figure 7:**
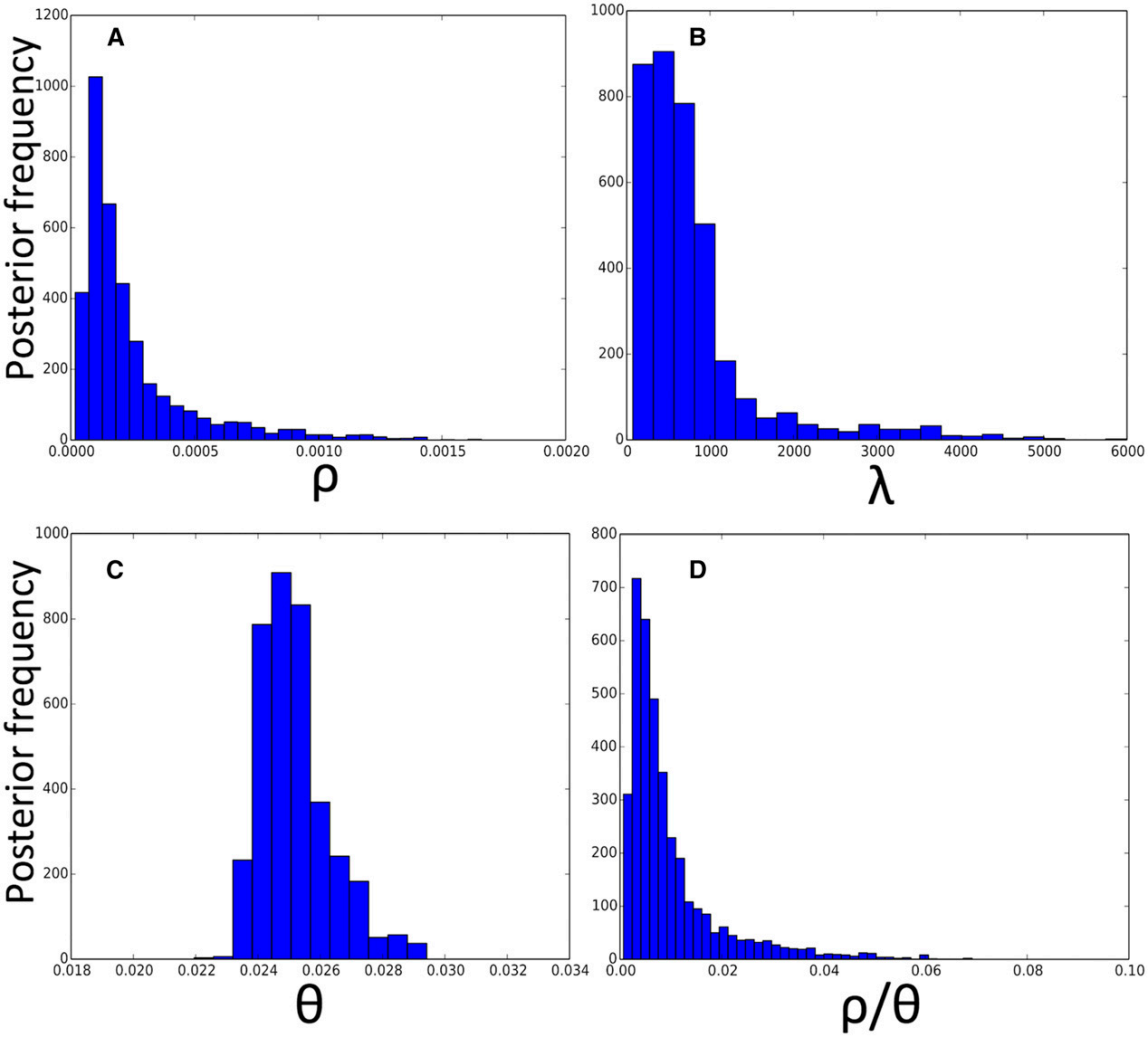
Posterior distributions of parameters for genome-wide evolution of *B. cereus*. We inferred BSMC parameters using an ABC-MCMC inference scheme. (A) Posterior distribution of *ρ*. (B) Posterior distribution of *λ*. (C) Posterior distribution of *θ*. (D) Posterior distribution of ρ/θ.

With our BSMC-based approach, we infer higher mean recombination tract length *λ* (median 592 bp and 95% confidence interval [119,3406], Figure 7) than previous estimates [171 and 152 bp from Didelot *et al.* (2010) and Ansari and Didelot (2014) respectively]; Our estimate is closer to values inferred from genome-wide likelihood-based analyses in *Clostridium difficile* (Didelot and Wilson 2015). We also inferred a considerably lower contribution of recombination relative to mutation (ρ/θ, median 0.0065 and 95 % confidence interval [0.001,0.038], Figure 7) than previous genome-wide studies [0.21 and ≈1.46 from Didelot *et al.* (2010) and Ansari and Didelot (2014), respectively]. This suggests that recombination contributes much less to *B. cereus* evolution than previously thought, and that these bacteria are considerably clonal, although, due to variation in recombination rates between clades, our results do not necessarily apply to all species in the *B. cereus* group (Sorokin *et al.* 2006). These results were confirmed by an additional independent run of the analysis (Figure S7 in File S1), and can be explained by the fact that we account for invariant sites, for transition/transversion bias, and for multiple substitutions at the same position (using a finite sites model). In fact, invariant sites and a high transition to transversion rate ratio usually cause more homoplasies than expected under a homogeneous substitution rate. This happens because an uneven distribution of substitutions along the genome (attributable to invariant sites) increases the probability that two substitutions hit the same base, potentially causing homoplasies. A transition/transversion bias increases the probability that bases hit by multiple substitutions become homoplasies. These homoplasies, if unaccounted for in the model, can be interpreted as the effect of short recombinant fragments, downwardly biasing estimates of *λ*, and upwardly biasing estimates of ρ/θ. Our approach naturally accounts for homoplasies due to multiple substitutions at the same site by modeling sequence evolution along local trees with a continuous-time DNA substitution model including invariant sites and transition/transversion bias. Supporting our interpretation, when we ran our method without accounting for invariant sites we estimated lower *λ* and higher ρ/θ (Figure S8 in File S1). Another potential factor is that our BSMC allows interactions between recombination events, breaking recombinant segments into smaller pieces as expected under the CGC; this process, if unaccounted for, could lead to a downward bias in the estimation of *λ*.

We can measure the total impact of recombination on genome evolution as ρ∗λ/θ, for which we infer a posterior median of 3.7 (95 % confidence interval [2.9,5.9]); this is considerably smaller than previous estimates [≈35.9 and ≈221.7 for Didelot *et al.* (2010) and Ansari and Didelot (2014) respectively]. Our ρ∗λ/θ confidence interval is smaller than for other parameters because *ρ* and *λ* are strongly inversely correlated in our posterior (Figure S9 in File S1). This makes ρ∗λ easier to estimate than the two parameters separately. This problem of identifiability of *λ* and *ρ* has been previously observed in bacteria (Ansari and Didelot 2014), and is also seen in eukaryotes (Padhukasahasram *et al.* 2004, 2006), although analysis of bacterial data are simpler due to the lack of crossover recombination. We found no correlation between other pairs of parameters (Figure S9, A–C, in File S1).

While our ABC-MCMC seems to capture well the complexity of real data for five out of seven summary statistics, for two of them (G4 at large distances and $r^{2}$ at short distances) there are discrepancies (Figure S9, D–J, Figure S7, E–K, in File S1). This suggests the existence of further neglected biological complexities, for example larger rate of recombination between closely related lineages (see Ansari and Didelot 2014), variable recombination rate between *B. cereus* clades (Sorokin *et al.* 2006), nonhomologous recombination (Didelot and Maiden 2010), population structure (such as due to niche adaptation Sorokin *et al.* 2006), recombination with other bacterial groups, variable selective pressure and mutation rate, and alignment errors. Error in clonal frame estimation, despite our efforts to correct branch lengths (see File S1), could also play a role in these discrepancies and reduce accuracy.

In conclusion, the BSMC offers not only a very computationally convenient approximation to the CGC, but also an accurate one. Our implementation of the BSMC model in the simulation software FastSimBac allows faster simulations of bacterial genome evolution (and therefore parameter inference with ABC), under a broader range of parameter values. FastSimBac allows specification of the clonal frame upon which simulations can be conditioned, which may grant simulations a closer fit to particular datasets when the clonal frame is readily estimable. By virtue of building on top of the popular simulators ms (Hudson 2002) and MaCS (Chen *et al.* 2009), our software includes options for many evolutionary scenarios that have been included in previous eukaryotic coalescent simulators (Hudson 2002; Chen *et al.* 2009), but which have remained unavailable for simulating bacterial genomes, such as population structure and migration, speciation, changes in population size, and recombination hotspots. Applications of our model and software are not restricted to simulations, but also include inference of recombination rates and other parameters of bacterial evolution. Our analysis of recombination in the *B. cereus* group showcases the applicability of our method for inference from genome-wide alignments. However, our ABC method is very computationally demanding, and so it would be challenging to apply it to scenarios with particularly high recombination rates, or large sample sizes. In the future, we intend to use the BSMC within a likelihood framework for accurate and efficient inference of the clonal frame and recombination parameters simultaneously. We believe that the BSMC and FastSimBac will prove very useful for both benchmarking and for statistical inference based on bacterial genome sequence data.

## Supplementary Material

Supplemental material is available online at www.genetics.org/lookup/suppl/doi:10.1534/genetics.116.198796/-/DC1.
