# Supplementary material for: The Bacterial Sequential Markov Coalescent
Source: Genetics. 2017 Mar 2;206(1):333–43. doi: 10.1534/genetics.116.198796 (PMC5419479; doi:10.1534/genetics.116.198796)
Supplement: Supplementary file 1 [file 333file001.pdf]

The bacterial sequential Markov coalescent

Nicola De Maio[1,2,*], Daniel J Wilson[1,2,3]

1 Institute for Emerging Infections, Oxford Martin School, University of Oxford , Oxford, United Kingdom
2 Nuffield Department of Medicine, University of Oxford, Oxford, United Kingdom
3 Wellcome Trust Centre for Human Genetics, University of Oxford, Oxford, United Kingdom

* E-mail: nicola.demaio@ndm.ox.ac.uk

Supplementary File S1

Algorithm for updating the ancestral material

We record ancestral material of each lineage as a list $m = (m_1, \ldots, m_n)$ of pairs $m_i = (s_i, p_i)$ with s_i integers and p_i real numbers with $p_0 = x_{cur}$ and $p_i > p_{i-1}$. The first element, m_1, tells us that from the current position $p_0 = x_{cur}$ until position p_1, the considered lineage is constantly ancestral to s_1 samples. The second element, m_2, if present, tells us that between positions p_1 and p_2 the considered lineage is constantly ancestral to s_2 samples. Similarly for the other elements of m. If for any $i \leq n$ we have $s_i = 0$ or $s_i = N$, where N is the number of samples, then the considered lineage is not in local tree between positions p_{i-1} and p_i. Here we discuss how we update in FastSimBac these ancestral material lists after the local ARG is modified by including a new recombination event and lineage. Updating the lists when x_{cur} is changed is instead trivial, as it just requires to remove all elements of the lists with $p_i \leq x_{cur}$. Also, there is no need to modify the lists after some lineages with no ancestral material are removed from the current local ARG.

The update of the lists is performed by a function addMRCAUp(l, m') that iteratively travels upward along the tree starting from lineage l, and updates the ancestral material lists of the lineages it encounters by summing ancestral material m' to it. addMRCAUp is called twice; the first time it is called on the new recombinant lineage l_1 with a positive m' (the ancestral material of the new lineage has to be added to the lineages above the one it coalesces to). If recombination happens on lineage l with ancestral material m, then m' is defined as the intersection of m with the recombining segment $m' = m \cap [x_{cur}, x_{end}]$ (m' has the same values as m on $[x_{cur}, x_{end}]$, and is 0 after x_{end}). addMRCAUp(l_1, m') is then called. If l_2 is the sister lineage of l_1 (the one with which it shares the recombination point), then addMRCAUp(l_2, $-m'$) is also called, where $-m'$ is obtained from m' by taking the same positions p_i but opposite counts $-s_i$.

Each call of addMRCAUp(l, m') does the following:

1 Add m' to the ancestral material of l.

2 If n_l, the node above l, is the root of the local ARG, end the iteration.

3 If n_l is a coalescent node with parent lineage l', call addMRCAUp(l', m').

4 Otherwise if n_l is a recombination node with recombinant parent lineage l_1 and sister l_2, and if $[p_1, p_2]$ is the recombining segment of n_l, then we will call addMRCAUp(l_1, $m' \cap [p_1, p_2]$), and addMRCAUp(l_2, $m' \setminus [p_1, p_2]$).

Where $m \setminus [p_1, p_2]$ is obtained setting all counts to 0 in m within the interval $[p_1, p_2]$. addMRCAUp(l, m') is not executed if m' is empty.

Features of FastSimBac

FastSimbac can be used to generate a sequence of local trees for subsequent intervals of the genome. For example local trees can be written to output with the option "-T":

```
./fastSimBac 10 10000 -r .001 500 -T 1>trees.txt
```

generates a sequence of local trees in the file "trees.txt" for a genome of 10000 bp, 10 isolates, a recombination initiation rate of $\rho = 0.001$, and a mean recombination tract length of $\lambda = 500$. SNPs can also be generated by specifying a mutation rate with option "-t", for example:

```
./fastSimBac 10 10000 -r .001 500 -t 0.002 1>sites.txt
```

simulates mutations along the local trees (generated similar to before) under a per-base scaled mutation rate of $\theta = 0.002$ and under a two-allele infinite sites model, and writes the list of SNPs generated in the file "sites.txt". For more realistic mutation scenarios, the local trees generated by FastSimBac can be used as input in SeqGen (Rambaut and Grassly, 1997).

Complex evolutionary histories of population structure and demography can be specified using options similar to ms (Hudson, 2002) and MaCS (Chen et al., 2009). For example, n populations with migration rate (backward in time) of $m = 0.1$ between them can be specified with the option "-I":

```
./fastSimBac 10 10000 -r .001 500 -I 3 5 1 4 0.1  1>output.txt
```

simulates 3 populations (with 5 samples from the first, 1 sample from the second, and 4 samples from the third) with migration rate of 0.1 (the migration rate for each population is 0.1 divided by the number of populations minus one). Population growth can be simulated with option "-G":

```
./fastSimBac 10 10000 -r .001 500 -G 0.3  1>output.txt
```

simulates under an exponentially growing ($\alpha = 0.3$ in this case, or shrinking if $\alpha < 0$) effective population size $N(t) = N_0 \exp(-\alpha t)$, where N_0 is the population size at time 0 (present) and t is the time before the present, measured in units of $2N_0$ generations. Further simulation options depending on a specific time t (such as population splits and merges) are described in the list of options at the end of this section.

We can also simulate recombination between populations or species, similar to Brown et al. (2015), but with some differences. Instead of modeling a generic diverged donor of recombining segments as Brown et al. (2015), we explicitly model different population/species and migration of recombinant segments between them. This makes simulations more realistic, as we consider the possibility of multiple donor species/populations with a given set of divergence times, and we also model the coalescent process of recombinant segments within a donor species/population. However, this also makes our model of inter-population recombination more computationally demanding. An example of usage is

```
./fastSimBac 10 10000 -x 0.0005 10 -T -I 2 0 10 0.0 -ej 20.0 1 2
```

which will simulate 10 samples collected all from the same population, a second unsampled population, no migration between the two populations (these 3 factors are specified by option "-I 2 0 10 0.0" as in ms), a merging backward in time of the two populations at time 20 (option "-ej 20.0 1 2" as in ms), and cross-population recombination rate 0.0005 and tract length 10 (option "-x 0.0005 10"). This scenario corresponds to two species diverged in the past, but which exchanged small pieces of the genome continuously after being diverged.

Option "-C" will condition simulations on the specified clonal frame. Leaves of the clonal frame Newick string must be named with integer numbers from 0 on. The specified clonal frame must contain branch lengths. An example of usage is:

```
./fastSimBac 3 10000 -r .001 500 -C "((0:0.1,2:0.1):0.2,1:0.3);"
```

Lastly, option "-R" instructs the software to use variable recombination rate as specified in an input file, for example to specify recombination hotspots and coldspots.

The full list of option is:

```
Usage: <samplesize> <region in base pairs> [options]
Options:
-s <random seed>
-d enable debugging messages
-i <iterations>
-t <mu> (mutation rate per site per 2N generations)
-C <clonalFrame> (the input clonal frame to fix simulations on,
sample names must be integers from 0 on)
-b <b> (burn in: sequence length in base pairs to be used to
initialize the recombination process)
-r <r> <lambda> (r = recombination (gene conversion) initiation
rate per-individual, per-base pair, per-2N generations; lambda =
mean length of recombination tract in base pairs)
-x <r2> <lambda2> (r2 = between-species recombination (gene
conversion) initiation rate per-individual, per-base pair, per-2N
generations; lambda2 = mean length of between-species
recombination tract in base pairs)
-T (Print each local tree in Newick format, compatible with
SeqGen, to standard output)
-F <inputfilename> [0|1] (Tab delimited frequency distribution
file where first column indicate range of SNP allele frequencies
from previous row to current row and last column is desired bin
frequency. Second parameter is 1 if SNPs with derived allele
freq > 1.0 should have alleles flipped, 0 otherwise)
-R <inputfilename> (Tab delimited file where first two columns
indicate range of base pair positions scaled to the unit interval
and last column is ratio with respect to base line recombination
rate)
-G <alpha> (Assign growth rate alpha across populations where
alpha=-log(Np/Nr)
-I <n> <n1> <n2> .. <mig_rate> (Assign all elements of the
migration matrix for n populations.  Values in matrix set to
mig_rate/(n-1) )
-m <i> <j> <m>  (Assign i,j-th element of migration matrix to m.)
-ma <m_11>..<m_12>..<m_nn> (Assign values to all elements
of migration matrix for n populations)
-n <i> <size>   (Pop i has size set to size*N_0)
```

```
-g <i> <alpha>  (If used must appear after -M option)

The following options modify parameters at time t.
-eG <t> <alpha>  (Assign growth rate for all pops at time t)
-eg <t> <i> <alpha>  (Assign growth rate alpha of pop i at time t)
-eM <t> <m> (Assign migrate rate m for all elements of migration
matrix at time t)
-em <t> <i> <j> <m_ij> (Assign migration rate for i,j-th element of
migration matrix at time t)
-ema <t> <n> <m_11>..<m_12>..<m_nn> (Assign migration rates
within the migration matrix for n populations at time t)
-eN <t> <size> (New pop sizes at time t for all pops where new
sizes = size*N_0)
-en <t> <i> <size_i> (New pop size of pop i will be set to
(size_i*N_0) at time t.
-es <t> <i> <p> (Split two populations.  At time t, a proportion p
of chromosomes from pop i will migrate to a population i+1)
-ej <t> <i> <j> (Join two populations.  At time t all chromosomes
migrate from pop i to pop j)
```

Correction of Branch Lengths in the ABC-MCMC Analysis

Let us assume that two samples have a time to most recent common ancestor (TMRCA) within the clonal frame of t, measured in number of $2N_e$ generations. Because of recombination, the local TMRCA at any site of the genome between these two samples might be different from t. For example, if the two samples are closely related, recombination events will likely cause their divergence to increase along some tracts of the genome. On the other hand, if the samples are distantly related, recombination might reduce their phylogenetic distance in some parts of the genome. More precisely, let us consider a given position of the genome, and let us denote with $P_2(x)$ the probability that at time x in the past (in units of $2N_e$ generations) the ancestral lineages of the two samples have not coalesced and are both in the clonal frame, with $P_1(x)$ the probability that one of them is in the clonal frame and one is recombinant, with $P_0(x)$ the probability that none of them is in the clonal frame and so both are recombinant and have not coalesced yet, and finally with $P_c(x)$ the probability that at time x the lineages have already coalesced. Then, we have by definition that $P_c(x) = 1 - (P_0(x) + P_1(x) + P_2(x))$, that $P_2(0) = 1$ and $P_0(0) = P_1(0) = P_c(0) = 0$, and that $P_2(x) = 0$ for $x > t$. Let us start by considering values of $x < t$. For these, given that the coalescent rate between any two lineages is 1, the following system of differential equations holds:

$$\begin{aligned}
dP_2(x)/dx &= -2\rho\lambda P_2(x) + P_1(x), \\
dP_1(x)/dx &= (-2 - \rho\lambda)P_1(x) + 4P_0(x) + 2\rho\lambda P_2(x), \\
dP_0(x)/dx &= \rho\lambda P_1(x) - 5P_0(x), \\
dP_c(x)/dx &= P_1(x) + P_0(x).
\end{aligned} \tag{S1}$$

At any value of $x > t$, the expected time to coalesce for the two lineages is 1. So the overall expected time to coalesce for two lineages is:

$$P_2(t)t + \int_0^t P_c(x)x dx + (P_1(t) + P_0(t))(t + 1). \tag{S2}$$

Equation S2 gives us the mean divergence that we expect to observe overall the genome between any two lineages given a clonal frame divergence of t. By assuming that the

genome is sufficiently large such that the observed divergence corresponds to the expected divergence, inverting this function, we can infer t. We performed this numerical integration to infer t both in R and Python and verified that we obtained consistent results. We show values of Equation S2 in Supplementary Figure S10. As can be seen, recombination pushes small values of divergence up, and large values of divergence down, overall homogenizing divergence among samples pairs.

Equation S2 expects, and returns, values expressed in terms of $2N_e$ generations, while what we can measure from data is only divergence in terms of genetic distance. To translate genetic distances into scaled time divergence, we assumed that recombination is strong enough so that the mean divergence time between all lineage pairs is 1, and we ignore recurring mutations.

Invariant Sites in ABC-MCMC analysis

We get a rough estimate of the transition/transversion rate ratio $\kappa = 5.21$ as the ratio of the number of observed biallelic SNPs involving a transition over the number of observed biallelic SNPs involving a transversion. We assume that P_0 is the proportion of invariant sites, and μ the transversion rate times genome-average total tree length. $G = 3610430$ is number of sequenced and aligned sites in the genome. Then we expect about $(1 - P_0)G\mu(2 + \kappa)$ biallelic sites and about $(1 - P_0)2\mu^2 G(1 + 2\kappa)$ triallelic sites along the genome alignment. Since we observe 556484 biallelic sites and 73090 triallelic sites, substituting these values in the previous equations, we get a back of the envelope estimate of $P_0 = 0.484$. While we are aware that our calculations are very approximate and that the concept of invariant sites itself is an approximation of the more realistic scenario of different degrees of selection affecting different sites, we only use these calculations here to test if accounting for selection can have a strong effect on the inference of recombination parameters.

References

T. Brown, X. Didelot, D. J. Wilson, and N. De Maio. Simbac: simulation of whole bacterial genomes with homologous recombination. *Microbial Genomics*, 2015.

G. K. Chen, P. Marjoram, and J. D. Wall. Fast and flexible simulation of dna sequence data. *Genome research*, 19(1):136–142, 2009.

R. R. Hudson. Generating samples under a Wright-Fisher neutral model of genetic variation. *Bioinformatics*, 18:337–338, Feb 2002.

A. Rambaut and N. C. Grassly. Seq-Gen: an application for the monte carlo simulation of DNA sequence evolution along phylogenetic trees. *Comput Appl Biosci.*, 13(3): 235–238, 1997.

Supplementary Figures

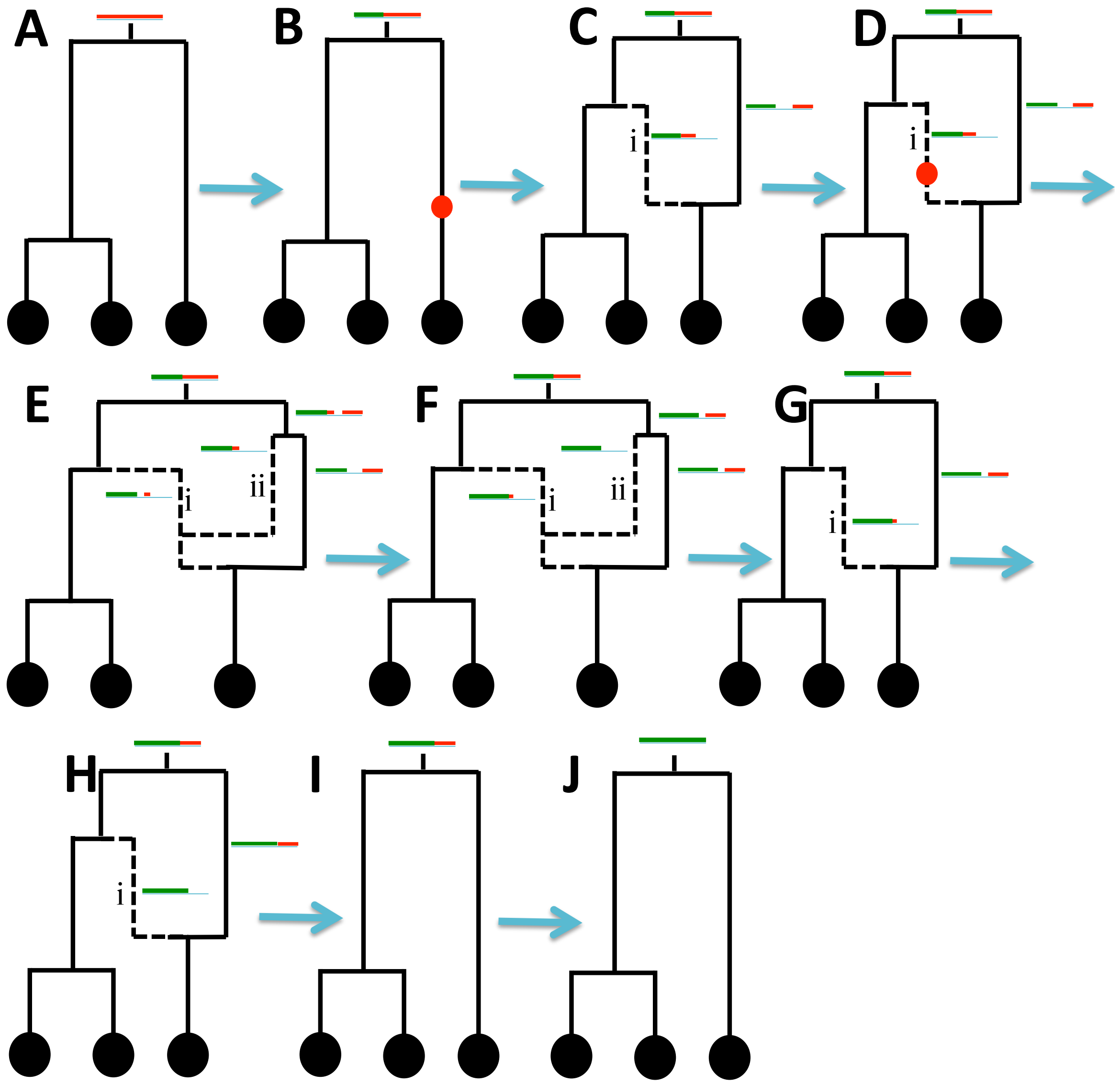

Figure S1. Graphical representation of the BSMC. Black circles represent samples, black lines are lineages (continuous if they belong to the clonal frame, dashed otherwise). Red segments represent, for each extant lineage, the portion of the genome that is ancestral to any sampled descendent of that extant lineage. Merges of lineages are coalescent events. The process moves left to right across the genome, and left portions of the genome are gradually forgotten (represented in green). **A)** The clonal frame is simulated at the left end (the start) of the genome; **B)** the first recombination event is sampled at the position of the red circle; **C)** the first recombining lineage is created (the dashed line, i) and is coalesced to the rest of the tree; **D)** a second recombination event is sampled (red circle), this time along a recombining lineage; **E)** the second recombining lineage is created (ii), and coalesced to the tree; **F)** the endpoint of the second recombination is reached, the second recombining lineage ii has no ancestral material left; **G)** the second recombining lineage ii is removed; **H)** the endpoint of the first recombination is reached, the recombining lineage i has no ancestral material left; **I)** the first recombining lineage i is removed; **J)** the right end of the genome is reached.

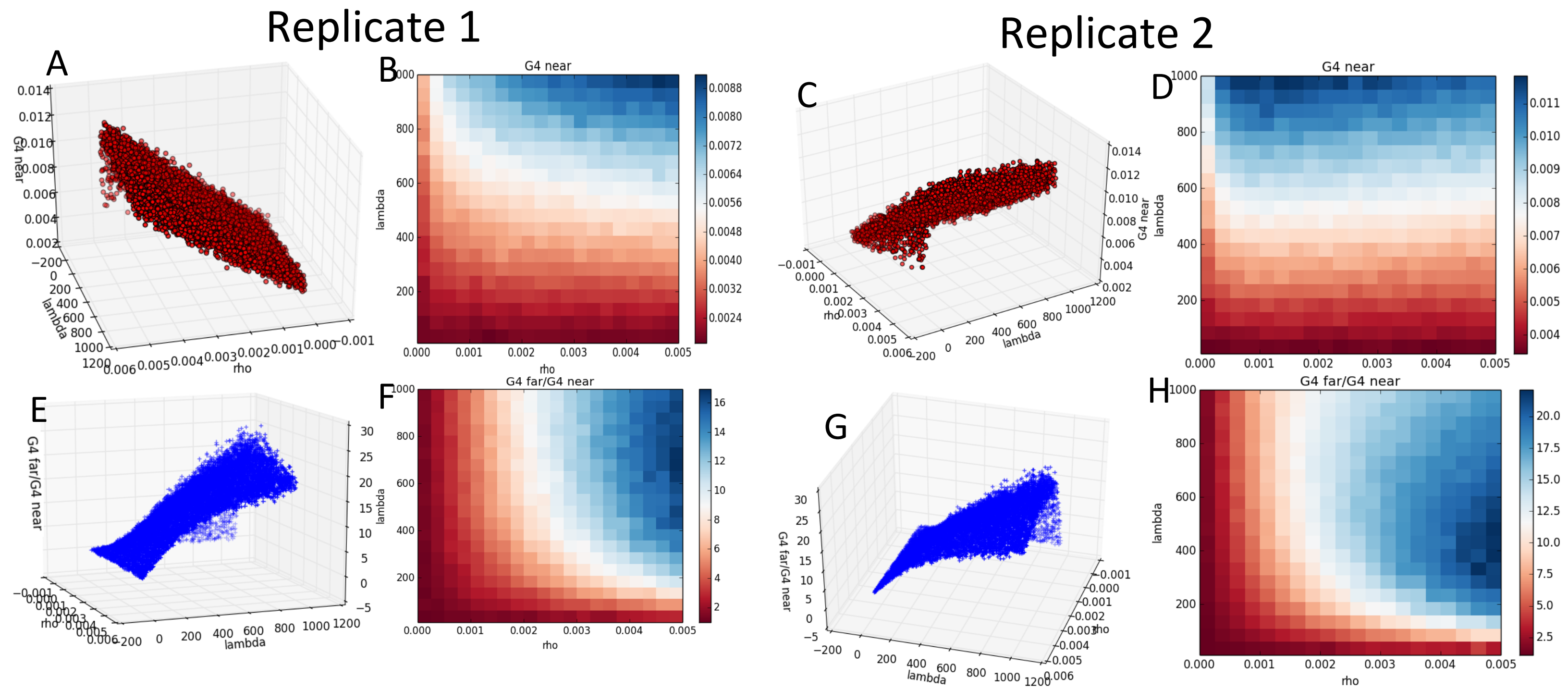

Figure S2. Summary statistics as functions of recombination parameters. Relation between summary statistics used in ABC simulations (G4 at short distance, first row, and G4 at long distance over G4 at short distance, second row) and recombination parameters. Values shown are those simulated for the ABC accuracy tests. 3D scatterplots (plots **A**, **C**, **E**, **G**) show values simulated as functions of ρ and λ (horizontal axes). Heat maps (**B**, **D**, **F**, **H**) show mean simulated values for binned combinations of ρ (X axis) and λ (Y axis). Left plots (**A**, **B**, **E**, **F**) show values for the first simulation; right plots (**C**, **D**, **G**, **H**) for the second simulation. Except for very small values of λ, G4 at short distance alone is very informative of the recombination rate. G4 at long distance over G4 at short distance is informative of lambda, except for low and high values of recombination.

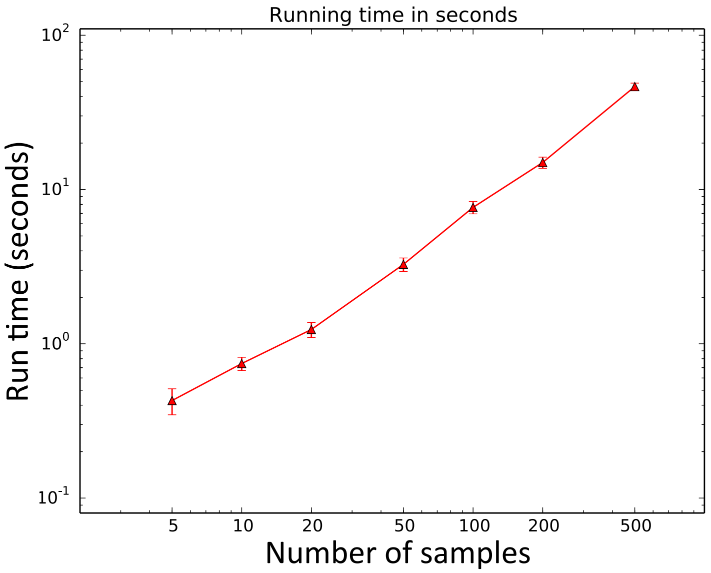

Figure S3. Computational demand of FastSimBac as a function of sample size. On the Y axis we show the per replicate running time of FastSimBac, expressed in seconds and on a logarithmic scale. On the X axis we show the number of samples simulated, on a logarithmic scale. Other parameters in FastSimBac are $\rho = 0.001$, $\lambda = 500$ and genome size 1Mbp. Each point is the mean over 10 replicates, and bars are standard errors of the mean.

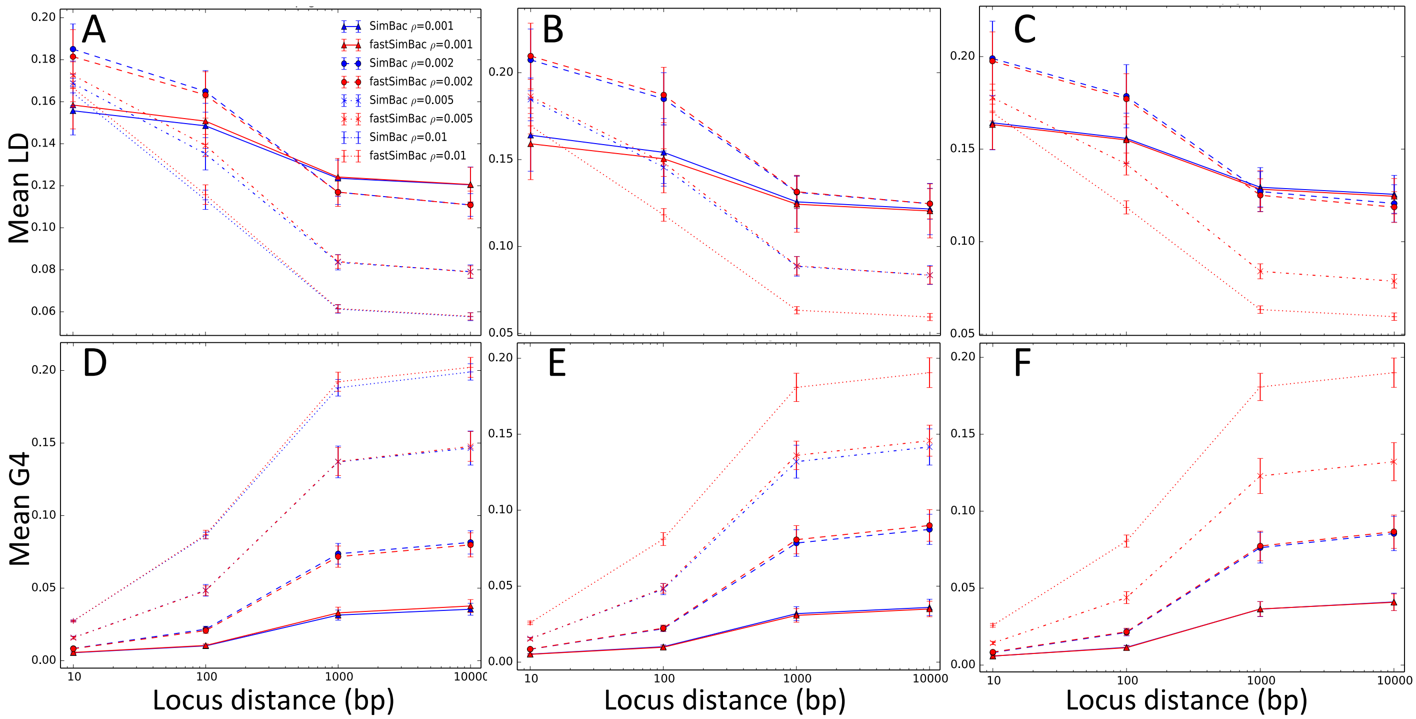

Figure S4. Comparison of linkage disequilibrium and site incompatibility patterns at longer genome sizes. The BSMC simulates patterns of linkage disequilibrium (LD) and site incompatibility (G4) very similar to the CGC. LD is calculated as $r^2 = \dfrac{(p_{AB} - p_A p_B)^2}{p_A(1-p_A)p_B(1-p_B)}$. G4 (the four-gamete test) is measured as the proportion of incompatible SNP pairs. On the X axis is the distance (in bp) between SNPs at which LD and G4 are calculated. For each distance d on the X axis, and for any SNP x in the alignment, LD and G4 are calculated between x and the first SNP at least d bp to the right of x. Red lines refer to FastSimBac, blue lines to SimBac, and different point and line styles refer to different recombination rates (see legend). Each point is the mean over 20 replicates, and bars are standard errors of the mean. **A)** Mean LD for a genome of 2Mbp, **B)** 5Mbp, **C)** 10Mbp. **D)** Mean G4 for a genome of 2Mbp, **E)** 5Mbp, **F)** 10Mbp. SimBac was not run for the highest recombination rates and genome sizes due to time limitations.

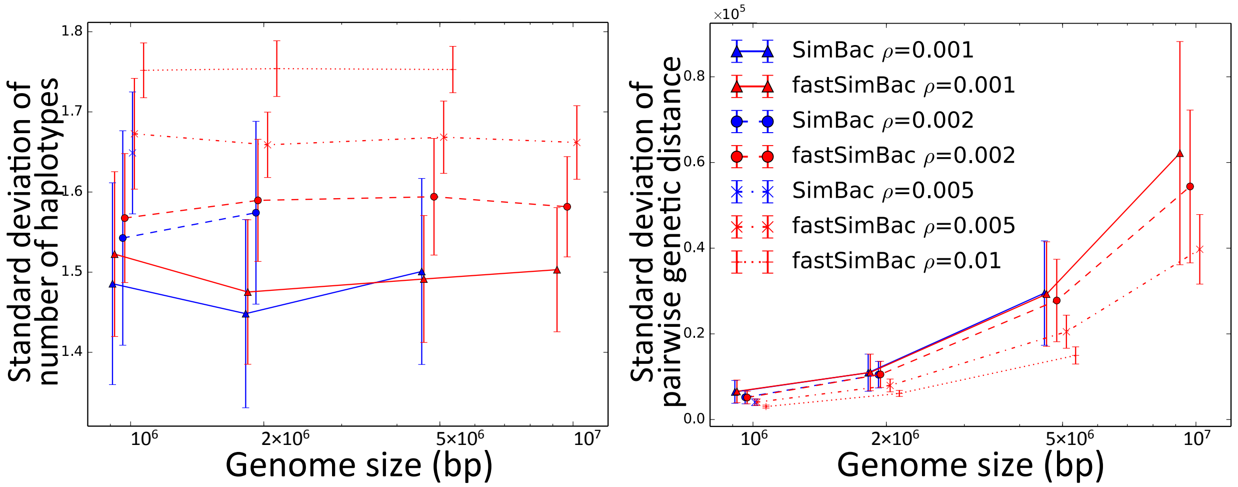

Figure S5. Comparison of genome-wide variation in genetic structure simulated by the BSMC and the CGC. The BSMC and the CGC show similar simulated variation in genetic structure along the genome. **A)** Genome-wide standard deviation of number of simulated haplotypes across non-overlapping sliding windows of 10 SNPs; higher values mean that different loci have more variable numbers of haplotypes. **B)** Standard deviation across sample pairs of the number of whole-genome genetic differences; lower values mean that genetic distances among sample pairs are more homogeneous (expected with higher recombination rates). On the X axis we show genome size in bp and on log scale. Red lines refer to FastSimBac, blue lines to SimBac, and different point and line styles refer to different recombination rates (see legend). Each point is the mean over 50 replicates, and bars are standard deviations. SimBac and FastSimBac were not run for the highest recombination rates and genome sizes due to time and memory limitations.

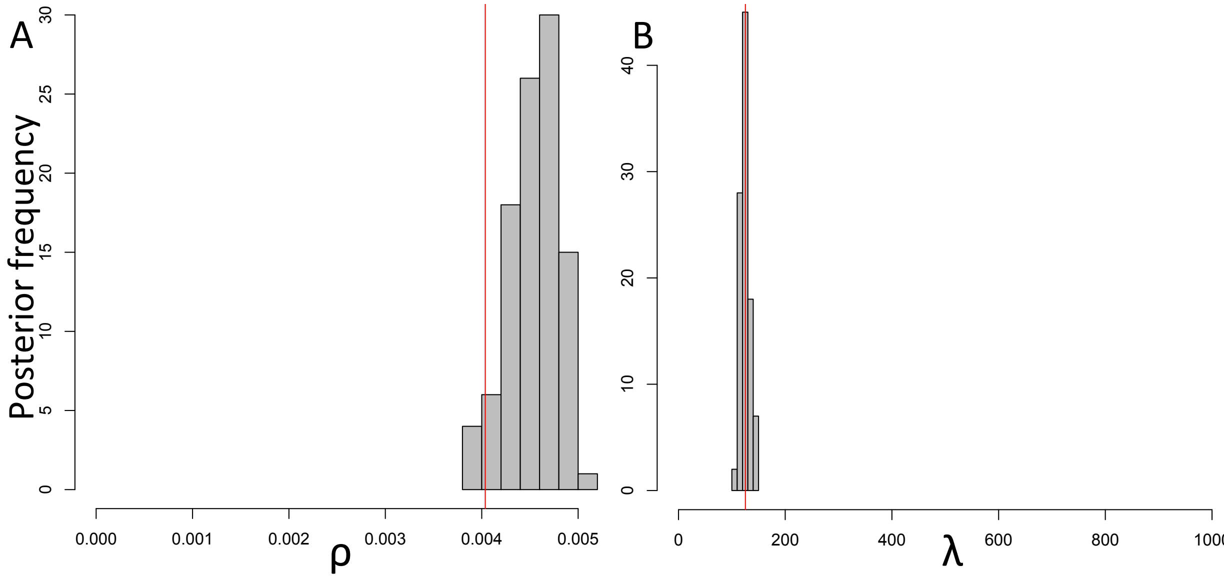

Figure S6. Second replicate of ABC inference using the BSMC on data simulated under the CGC. Recombination parameters simulated under the exact CGC (red vertical lines) where reconstructed using simulations under the BSMC within an ABC inference scheme. **A)** Posterior distribution of ρ. **B)** Posterior distribution of λ.

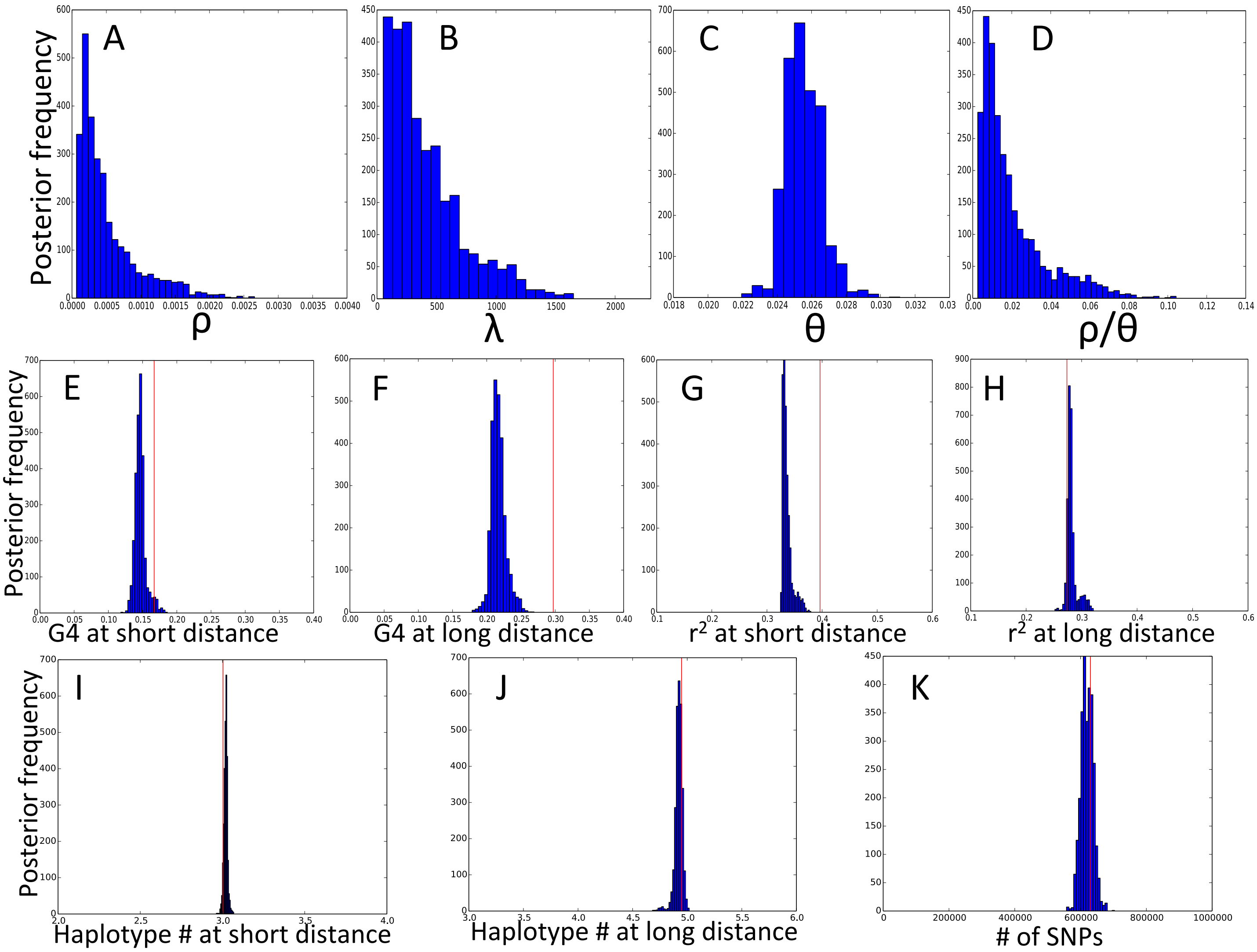

Figure S7. Posterior distributions of parameters and summary statistics for a second inference of genome-wide evolution of *B. cereus*. We inferred BSMC parameters using an ABC-MCMC inference scheme, similar, but independent, from the one in Figure **??** and Supplementary Figure S9. **A)** Posterior distribution of ρ (interquartile range $[0.0002, 0.0007]$). **B)** Posterior distribution of λ (interquartile range $[181, 563]$). **C)** Posterior distribution of θ (interquartile range $[0.0247, 0.0262]$). **D)** Posterior distribution of ρ/θ (interquartile range $[0.008, 0.026]$). **E)** Posterior distribution of G4 (proportion of SNP pairs breaking the 4-gamete rule) for consecutive SNPs (short distance); **F)** Posterior distribution of G4 for SNPs at least 2kbp away (long distance); **G)** Posterior distribution of mean linkage disequilibrium (LD, measured as r^2) for consecutive SNPs (short distance); **H)** Posterior distribution of mean linkage disequilibrium for SNPs at least 2kbp away (long distance); **I)** Posterior distribution of mean number of haplotypes for pairs of consecutive SNPs (short distance); **J)** Posterior distribution of mean number of haplotypes for groups of 4 SNPs made of 2 pairs of consecutive SNPs, the two pairs being at a distance of at least 2kbp (long distance); **K)** Posterior distribution of number of SNPs. Summary statistics of the real dataset are shown with red vertical lines in plots **E-K)**.

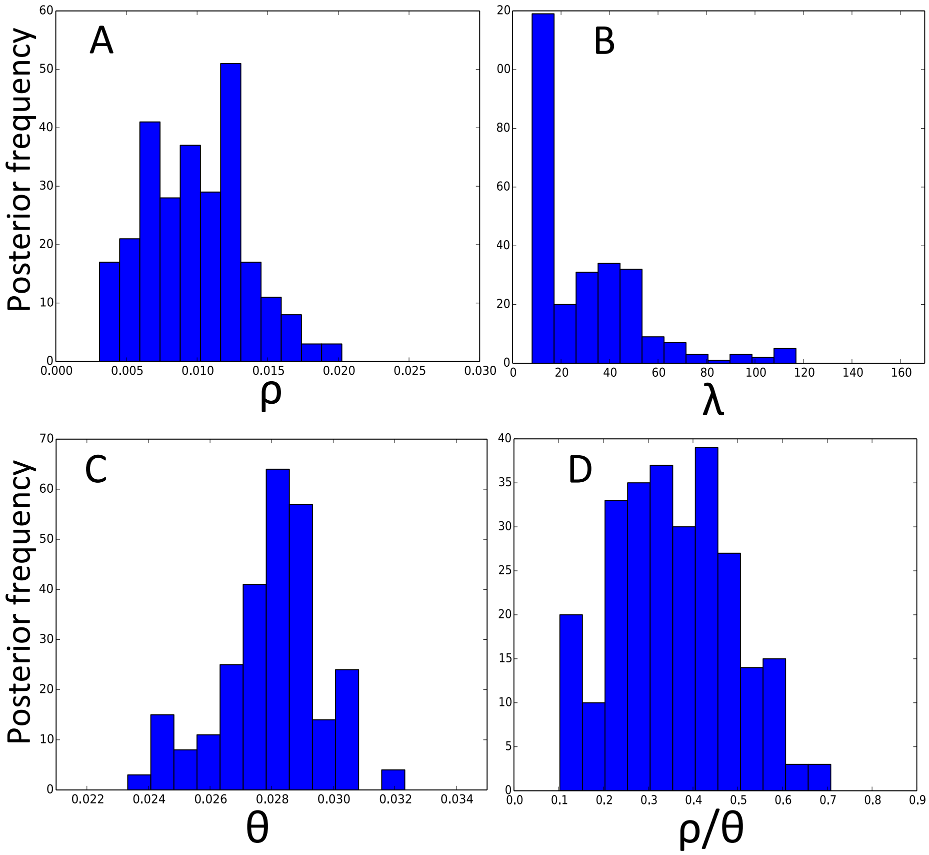

Figure S8. Posterior distributions of parameters for genome-wide evolution of *B. cereus* when not accounting for invariant sites. We inferred BSMC parameters using an ABC-MCMC inference scheme as in Figure **??**, but this time without account for invariant sites, without correcting branch lengths, and using 5000 ABC-MCMC steps. **A)** Posterior distribution of ρ (interquartile range $[0.007, 0.013]$). **B)** Posterior distribution of λ (interquartile range $[14, 44]$). **C)** Posterior distribution of θ (interquartile range $[0.0271, 0.0289]$). **D)** Posterior distribution of ρ/θ (interquartile range $[0.257, 0.449]$).

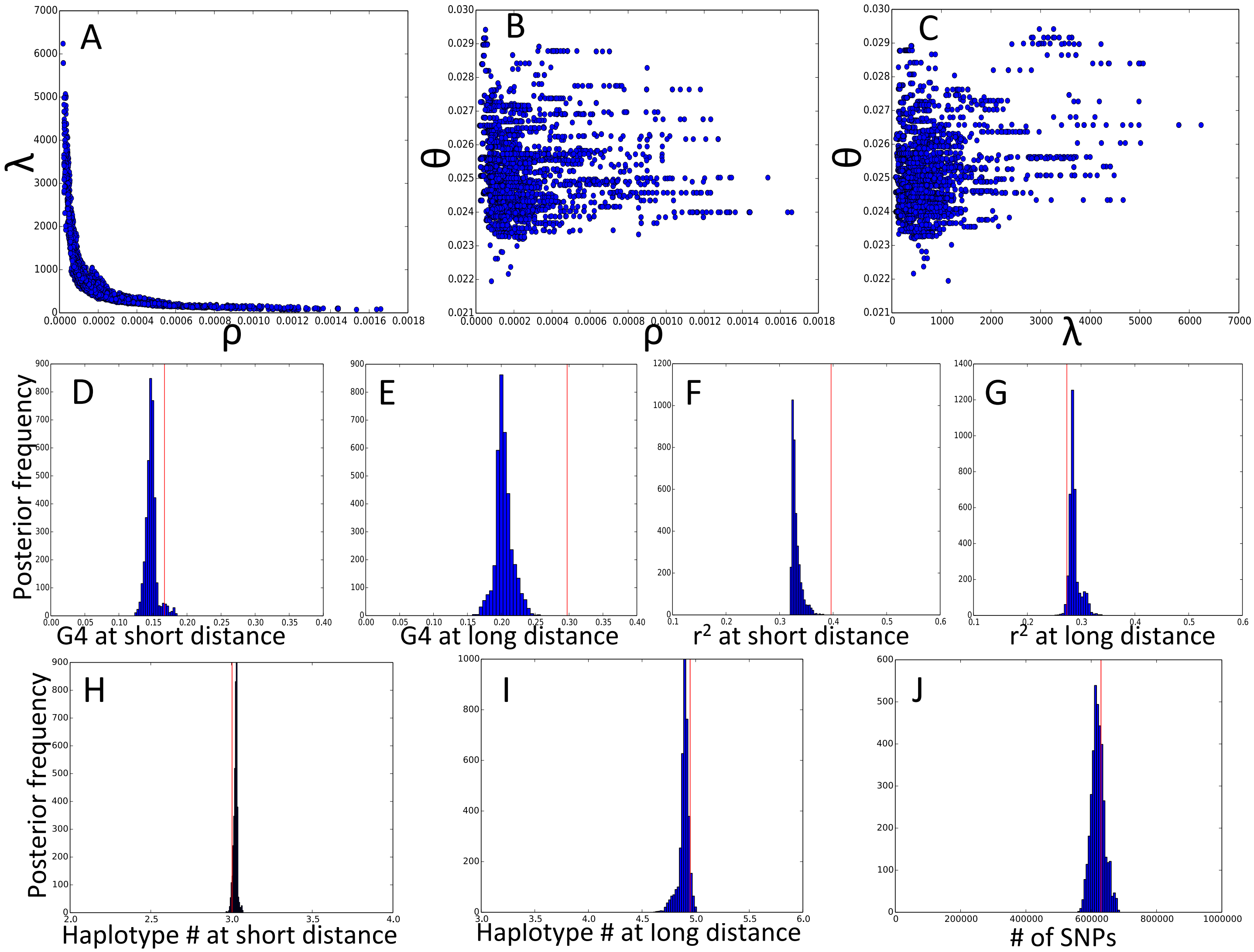

Figure S9. Scatterplots of posterior parameters and summary statistics for genome-wide evolution of *B. cereus*. We inferred BSMC parameters using an ABC-MCMC inference scheme. In the first three scatterplots we show posterior distributions of **A)** ρ and λ; **B)** ρ and θ; **C)** λ and θ. In the second and third rows we show the posterior distributions of summary statistics: **D)** G4 (proportion of SNP pairs breaking the 4-gamete rule) for consecutive SNPs (short distance); **E)** G4 for SNPs at least 2kbp away (long distance); **F)** mean linkage disequilibrium (LD, measured as r^2) for consecutive SNPs (short distance); **G)** mean linkage disequilibrium for SNPs at least 2kbp away (long distance); **H)** mean number of haplotypes for pairs of consecutive SNPs (short distance); **I)** mean number of haplotypes for groups of 4 SNPs made of 2 pairs of consecutive SNPs, the two pairs being at a distance of at least 2kbp (long distance); **J)** number of SNPs. Summary statistics of the real dataset are shown with red vertical lines in plots **D-J)**.

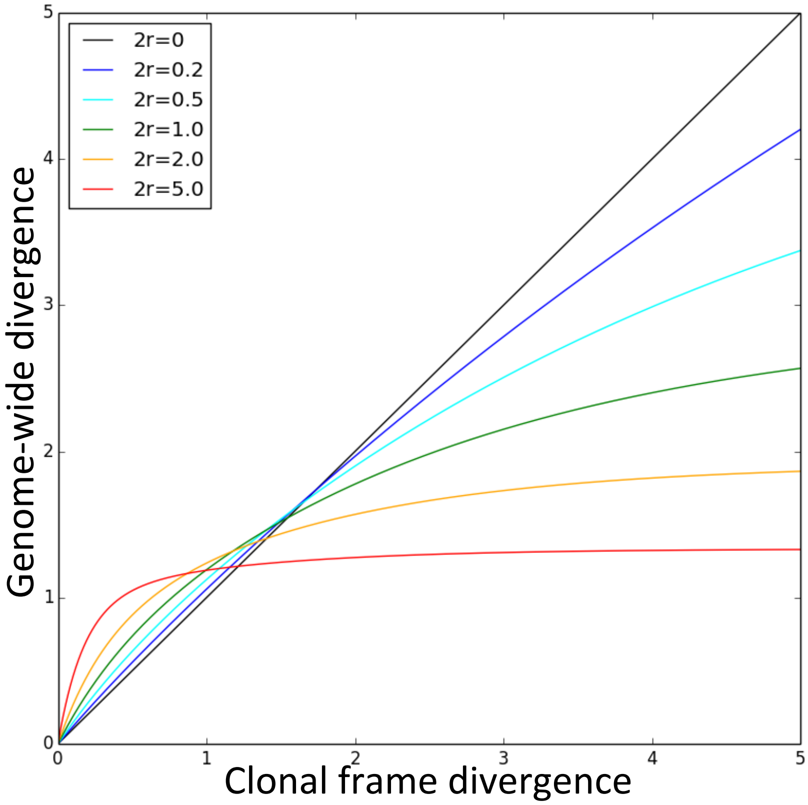

Figure S10. Effect of recombination on mean divergence between pairs of samples. Different line colors represent different recombination rates (from $\rho = 0$ to $2\rho = 5$) as in the legend. On the X axis is the clonal divergence of two samples. On the Y axis is the genome-wide mean expected divergence accounting for the effect of bacterial recombination. In the absence of recombination, clonal divergence and genetic divergence are perfectly correlated (black line). The higher the recombination rate, the more genetic divergence flattens. Values shown are obtained with equation S2.
